# On the Operation for Empyema

**Published:** 1842-04

**Authors:** 


### ART. IV.

1. *De l'Opération de l'Empyème.* Par M. C. SEDILLOT, Chirurgien-major, Professeur à l'Hôpital du Val de Grace, &c.—*Paris,* 1841. 8vo, pp. 181.

*On the Operation for Empyema.* By M. C. SEDILLOT, Surgeon to and Professor at the Val de Grace Hospital, &c.—*Paris,* 1841.

2. *Notes on the Treatment of Chronic Pleurisy, with Effusion.* By the late JAMES HOPE, M.D.—*London,* 1841. 8vo, pp. 8. (*From the Medico-Chirurgical Review,* No. LXIX.)

3. *The Article "Empyema."* By Dr. WALSHE. (*Cyclopædia of Practical Surgery,* Nos. IX-X.)—*London,* 1841. 8vo.

THESE essays were produced with different objects: M. Sédillot undertakes simply to canvass the merits of, and establish the best mode of performing the operation for empyema; Dr. Hope, to describe such a medical treatment as shall make the operation unnecessary; Dr. Walshe, to supply a complete history of empyema, as far as the arrangement of the work for which he has written permits this.

These writers use the term empyema in the practical, and not in the etymological sense; understanding by it, to use the words of one of them (Dr. Walshe), "every fluid pleuritic collection, whether purulent, puriform, albumino-serous, or otherwise, which runs a manifestly chronic course, and continues stationary, or increases in spite of remedial measures." To us this appears perfectly in accordance with necessity; so long as the state of knowledge continues such, that the nature of the liquid cannot during life be ascertained, the word can only be used thus, if it be used at all. "The term," observes Dr. Walshe, "is evidently an incorrect one; but as its conventional meaning is generally understood, there is perhaps no adequate motive for changing it." From the inability just referred to it follows that all attempts to describe empyema without reference to pleurisy, tending as they must to generate the notion that it commonly arises as a perfectly independent disease, must give the student a false idea of its pathology. By briefly but carefully tracing the history of pleuritis, Dr. Walshe has given a degree of precision to the subject of empyema which, as it is usually handled in surgical works, it signally wants. We shall, however, not enter into this part of the subject here, as we are desirous of confining ourselves as much as possible to the consideration of empyema practically. Having shortly noticed the statements in one of the productions before us respecting the mode of increase of effusions into the pleura, and borrowed an extract from its section on the diagnosis of those affections, we shall pass to the subject of treatment.

Dr. Walshe divides the stage of effusion into three sub-stages; those of laminar effusion, gravitating effusion, and effusion with dilatation of the chest and "*detrusion*" of the surrounding parts. We confess that on first view this appeared to us a piece of hyperdivision, but upon enquiring into the grounds of the arrangement, we found not only that it is perfectly warranted by observed facts, but is one involving practical considerations of no mean importance.

"While the liquid accumulated," observes the author, "is moderate in quantity, this is found (where the physical principles carefully considered by M. Woillez would prepare us to find it) equally spread over the entire pulmonary surface (laminar effusion). This disposition of the fluid will continue so long as the aspiration of the lung exercises a more effectual influence upon the position of the fluid, than its own gravity. With the continuance of effusion the force of gravity becomes predominant and the fluid accumulates at the most depending part of the chest (gravitating effusion). The duration of the first stage is of course mainly regulated by the activity of effusion; but is also to a less degree affected by the degree of elastic contractility of the lung."

While a thin structure of fluid of equal or very nearly equal thickness in all directions is spread over the entire surface, the sound on percussion is less clear than natural, but is not completely dull: the loss of sonorousness is proportionably equal in all parts of the surface; there is, therefore, no precise limitation between a dull and a clear sounding part; nor are the position or other characters of the dull sound capable of being altered by changing the position of the patient. This form of effusion is not, we believe, habitually recognized by writers, but we believe we have seen cases which gave the warranty of experience to what appears on physical grounds so fairly admissible. These cases were of the kind alluded to by Dr. Walshe in the following passage:

"When liquid has collected in sufficient abundance to overcome by its gravity the other physical force acting upon it, the signs change. The upper part of the chest recovers in a great measure or even completely its normal sonorousness: hence a diminution of the extent over which dulness is perceptible actually announces increase of the disease. Though rare, we have observed a few examples of this occurrence; practically it is of much importance, for it might easily betray the observer into a belief that absorption had commenced. The inferior part of the chest of course sounds duller than before; but, at first, this increase is not strongly marked. The dull sound is now limited superiorly by a well-defined line answering to the upper border of the effusion; and the limits of the dull sound may be changed by causing the patient to assume different positions, inasmuch as the lung floats on the surface of the fluid, while this collects in whatever part of the chest the changing position of the patient renders the most dependant."

At length fluid accumulates to such extent as to destroy the elasticity of the lung completely, the organ is incapable of further retreat before the increasing effusion, consequently this acts on the walls of the chest and the surrounding soft parts; we have now the stage of effusion with dilatation and "*detrusion*" of organs. This being the most common condition of things in empyema is dwelt upon particularly by Dr. Walshe in respect of its physical signs; and certainly with the abundance of means of information here exhibited, to guide us in our diagnosis, error would appear to be hardly venial.

One of the most important points connected with the history of empyema in the stage of absorption with contraction, is the position of the heart. The following different conditions have been observed: 1. The organ recovers its normal position, or the immediate vicinity of this. 2. In certain cases of empyema of the right side it is drawn during the progress of absorption into the right division of the thorax, (Stokes, Williams). 3. The heart on account, it is presumed, of loss of tone on the part of the mediastum, acquires a certain share of mobility, and falls to the right when the patient lies upon or leans to that side. A single example of this state has been observed by Dr. Stokes. 4. "The heart remains in the abnormal position into which it was forced by the effusion, in consequence probably of the establishment of adhesions." A priori considerations would lead us to suppose this condition to be far from uncommon, yet we do not remember to have seen it noticed before: Dr. Walshe gives a figure of the thorax of a subject in whom he observed it.

Dr. Walshe institutes a very minute enquiry into the differential signs of empyema and cancerous accumulation in the pleura, but it is too long for extraction, and we are unwilling to abridge.

The views promulgated by Dr. Hope in the paper of which we have given the title are of a very encouraging character, as regards the possibility of curing this formidable disease. We think we cannot do better than adopt the account of his practice given by Dr. Walshe:

"Dr. Hope conceives that the great mortality which he ascribes to chronic pleurisy, in some measure depends upon the error of systematic writers, 'who mistake the state of anæmia, with its quick pulse, for irritative fever, by which they not only mislead themselves, but also their readers, as to the nature of the patient's condition, and consequently as to the appropriate means of cure.' Hence has arisen, according to Dr. Hope, a far too unfavorable impression of the curability of chronic pleurisy with effusion. He had himself been 'instrumental in curing five and thirty cases consecutively, during the space of four years; but principally two years and a half, while assistant-physician to St. George's Hospital.' The treatment under which these favorable results occurred was as follows: Dr. Hope always used mercury 'in all degrees of intensity, with opium in full proportion,' the milder and external forms when the others could not be borne, and to such extent as to produce prompt and free salivation. Blisters, with diuretics—squill, nitric æther, juniper, iodide of potassium, and, when there was no irritation of the mucous membranes, the various other preparations of potass were conjoined with the mercurial treatment. A powerful hydragogue was had recourse to, if the remedies now enumerated had failed, for two or three days: half a grain to a grain of elaterium, combined with calomel and capsicum to prevent nausea; or the pulv. jalap. comp., so as to produce ten or twelve copious watery evacuations per diem, stimulants being at hand in case of any sinking tendency.

"Inconvenience sometimes resulted from hypersalivation, which greatly retarded the convalescence. The action of the remedy was, however, easily controlled, by omitting it for two or three days, or by merely diminishing the dose: it did not do to suspend its use altogether. Much stress is laid by the author upon the statement, that great acceleration of pulse, with thirst, craving for cold drinks, and dry heat of skin, is not necessarily a result of fever, but *is* necessarily a result of anæmia, occasioned by the deficiency of oxygenation from the total incapacity of one lung at least. To patients thus conditioned, Dr. Hope allows at least one or two pints of concentrated beef-tea; and, if the state of the tongue and alimentary canal fully authorize it, permits them tender old mutton or beef for dinner. Even when hectic fever is established, Dr. Hope found the use of mercury still indispensable; when it had been omitted contrary to his wishes, a recurrence of the effusion has taken place, and on the resumption of mercury with opium the fluid has again disappeared. When the hectic fever is nearly removed, preparations of iron and mineral acids are given to cooperate with nourishing food in removing all remains of anæmia. These latter medicines are to be well guarded with laudanum."

Dr. Walshe recognizes three objects which should be kept steadily in view in conducting the medical treatment of empyema: 1. The prevention or controlling of febrile action. 2. The promotion of absorption. 3. The support of the general health. 1. He is an advocate for general bleeding, "if the antiphlogistic plan have not been put in force with sufficient energy during the acute stage; if febrile action, accompanied or not with local pain, exist in a subject labouring under chronic pleurisy; and if the constitutional powers appear not to be seriously depressed." But even under these circumstances he inculcates the observance of extreme caution. "An amount of depression will occasionally follow very moderate loss of blood from a vein, which may not easily be recovered from," even in subjects apparently the best suited to undergo such evacuation. Against the employment of local bleeding, either by leeching or cupping, Dr. Walshe thinks no such objections hold good, and would apply six or eight leeches to the affected side two or three times a week for say a fortnight, "and this even in cases where febrile action and local pain are almost or totally absent, provided the effusion be not of old standing," in which case he has not observed much benefit to follow local depletion. These views are not novel, but they seem to us of sufficient importance to justify their being enforced by repetition. This author thinks highly of the exhibition of calomel and opium; but would exclude it from decidedly chronic asthenic cases, and those in which the effusion is purulent: herein, if at variance with Dr. Hope, he has Professor Williams on his side.

2. Among agents promotive of absorption, Dr. Walshe appears to have particular confidence, as diuretics, in *infusion* of digitalis and iodine: we believe that the utility of the latter in these cases is more generally recognized. The following observations on the sustained use of *purgatives* are practically important:

"Purgatives should be very cautiously exhibited when the lungs are tuberculous, either because the intestinal tunics already contain tubercle, which may thereby be more speedily brought into the stage of softening, and induce ulcerative destruction, with its consequences, of the mucous tunic; or because the irritation of the bowel in a subject already phthisical accelerates the deposition of tubercle there. We have seen at least one case in which uncontrollable diarrhœa, brought into existence under such circumstances, evidently hastened the patient's dissolution. On the other hand, dyspnœa, which has resisted all endeavours for its relief, may occasionally be removed or materially relieved, and this almost instantaneously, by an active hydragogue. We have observed this from the exhibition of elaterium, even where no manifest change in the thoracic physical signs followed."

Upon the importance of repeated application of blisters, Dr. Walshe forcibly insists, he is "the more anxious to state emphatically their utility in these cases; because the very justifiable incredulity as to their *therapeutical* efficacy in acute parenchymatous and membranous inflammations, now arising among the profession, might possibly be extended to their action in chronic maladies of the kind at present under consideration."

3. After some observations upon the necessity of supporting the general health in advanced cases by succulent diet, gentle tonics, and change of air, Dr. Walshe passes to the surgical treatment of the disease; and this leads us to examine the work of M. Sédillot. "The operation for empyema," says M. Sédillot, "is generally imperfectly understood, ill-performed, and ill-appreciated," a state of ignorance which he evidently conceives himself destined to put an end to altogether. "It would be an error, however, to believe," observes this modest reformer, "that we denounce *all* that has been hitherto done on the subject, and come forward to *create* the operation for empyema." Unheard of forbearance! And this, too, from a surgeon, who, if we may judge from his book, and he is not likely to have hid his light under a bushel, has never performed the operation, while it is equally plain that his 181 pages contain not one single indubitably-novel suggestion. He believes himself, however, to have all but "created the operation," because, having collected together a number of cases and fancied them to furnish motive for a certain line of conduct, he publishes the whole in a goodly octavo! But while we castigate the absurd self-complacency with which this writer magnifies his mole-hill into a mountain, we would not deny his production merit—much merit, though of a kind infinitely more humble than that wherewith himself is persuaded it abounds. In a first part is contained an examination of ancient and modern doctrines upon the operation; in a second, are printed in goodly type, each followed by sundry reflections, forty-nine reports of cases obtained from various French works, of the utility of both these parts we are quite as well persuaded as their author. In a third part, "for which the first two will have served as introduction and justification, are traced the general rules for the operation;" of the extreme value and accuracy of the doctrines laid down in this part we shall probably, as we advance, be inclined to indicate our suspicions. We shall pass over the first two parts, confining our notice of them to such references as are made requisite by our examination of the third; and this latter will only come under review in respect of empyema properly so called; to enter upon the subject of paracentesis for the relief of hydrothorax and pneumothorax would carry us beyond our limits.

M. Sédillot first enquires whether the operation is an admissible one, a query which we shall answer in the words of Dr. Walshe.

"Under these circumstances, a resource is still left in the operation of paracentesis, a procedure which, no matter how divided opinion may be respecting its general feasibility, has assuredly been sufficiently often either completely successful or productive of marked improvement, to justify its being numbered among the valuable gifts of surgery. This is a vague estimate of its utility, it may be alleged; but we fear such vagueness of expression is unavoidable. In truth, there are no existing data from which precise inferences may be drawn as to the success of this measure, whether considered generally or in reference to its performance in particular states of the constitution. And this because observers have contented themselves with merely ascertaining the existence of effusion into the pleura of subjects operated on, without enquiring into the condition of other organs, and above all, substantiating the presence or absence of pulmonary tuberculization. How can a correct general inference be obtained, when patients are clubbed together, who have simple chronic pleurisy, or this combined with carious destruction of the ribs; who have or have not serious organic disease in other regions of the body; whose lungs are sound, or the seat of active tuberculous disorganization?"

In this passage Dr. Walshe appears to us to have justly stated the difficulty of forming any precisely accurate notion of the amount of good to be effected by the operation. There are not sufficient facts on record —facts unselected, of success, and of the contrary, accurately and fully reported—to admit of the sound application of the numerical method. Now were we called upon to point out the practical question most imperatively calling for the elucidation of that method we should probably indicate the very one now before us, as being that in which all attempts at settlement based upon argument and ordinary synthetic reasoning had failed in the most irretrievably hopeless manner. Let him who doubts this, peruse the report of the ludicrous—we know no other word to describe it by—discussion which took place on the subject at the Paris Academy of Medicine in 1836; in which some of the learned academecians exhibited, by the way, such steadiness of belief and depth of conviction that before its conclusion they actually changed their opinions *in toto* on some of the most important topics mooted during the course of the debate. And the contradictory opinions maintained by the profession at Paris upon this memorable occasion had all of them their warranty in facts, ill-observed ones, no doubt, but still facts. They who would have the opening in the chest *closed* after the operation, for example, could cite their cures when such closure had been made, and gibe their adversaries with the deaths which had followed where it had been neglected; but their triumph lasted but a moment, for behold an advocate for the plan of keeping the orifice carefully *open* is upon his legs and recounts in set phrase to the puzzled auditory how such and such patients manifestly owed their recovery to that mode of treatment, and how others as assuredly had died, in whom a sufficient vent had not been established for the accumulating fluid. Now, to us, M. Sédillot appears to have done nothing more than any of these debaters: partly guided by the results of a very limited number of cases, and much more truly actuated by theoretical considerations, he advocates a certain mode of operating, forgetting that patients treated in those very manners which he strenuously reprobates, have recovered in equally great, perhaps proportionably greater numbers. But we anticipate.

The first point to be decided is the situation in which the opening is to be practised. The common habit of surgeons has been to operate at the most dependent and prominent spot of the antero-lateral portion of the chest, that is between the third and fourth false ribs on the left side, and between the fourth and fifth on the right. Dr. Walshe agrees with Dr. Stokes in believing that no real advantage is secured by the performance of the operation in these spaces, and that a risk is thereby incurred—no imaginary one, as numerous unfortunate occurrences of the kind on record prove—of wounding the diaphragm and abdominal viscera. The establishment of a high opening is supported by analogical reasoning. Nature, whose performance of paracentesis is generally more successful than that by the surgeon, commonly perforates the chest by ulceration in or above the fourth or fifth intercostal space. M. Cruveilhier, it appears from the statement of M. Sédillot, also inclines, on the ground of this fact, to the performance of the operation between the fourth and fifth true ribs: the latter surgeon, for reasons which appear to us of trivial importance, would open the chest inferiorly and posteriorly.

What quantity of the contained fluid is to be evacuated at the moment of the operation? enquires M. Sédillot. The query is replied to as follows: "The indication is to remove the liquid in such quantity as may be proportional to the power possessed by the surrounding organs of supplying its place." Complete evacuation he would confine to cases in which the lung is not invested by pseudo-membrane, or atrophous, and more especially to those in which the pleura is not seriously diseased: examples of hemothorax are referred to in the latter clause. There is, however, a palpable objection to partial evacuation which does not appear to have impressed M. Sédillot sufficiently strongly; little or no relief is afforded until the whole or by far the greater part of the fluid is removed: patients have been known to sink into a sate of utter despondency upon finding themselves in as much misery after as before the operation in cases where either the will of the surgeon, or some peculiarity in the condition of the pleura prevented the free egress of the entire accumulation. When this has been accomplished, a state of perfect ease, comparatively so at least, has followed almost instantaneously. The real question, however, is which plan can boast of the greater number of recoveries: Dr. Walshe states that the practice of those who empty the sac at once, as completely as possible, has been the most successful; and in this statement our own reading would lead us to concur.

The manner of regulating the flow of liquid after the first evacuation is next discussed by M. Sédillot. The views he holds upon this point appear to him of the least consequence, it may be therefore well to set down his motives for the entertainment of them. "It is impossible that adhesion can be effected between two parts which having been for a moment only approximated to each other, are kept continually asunder; hence the necessity of successively diminishing the quantity of the pleural fluid. Our object must be to favour the escape of any new matter which is secreted, and besides to remove a small portion of that left in the suppurating cavity, so as to allow this to diminish in extent and yield to the concentric movement of the neighbouring organs." Such are the indications; but as they are beyond our reach now, according to M. Sédillot, all we have to do is to effect nearly as possible their fulfilment. "The cicatrization of the wound is to be prevented, and the opening used occasionally for the discharge of the remaining fluid, so as to obviate the risk of renewed distension; there would be no objection to allowing the fluid to ooze away gradually, *if* it were possible at the same time to prevent the entry of air into the chest." Doubtless, there would be no such objection *if* the occurrence of pneumothorax could be prevented; but it is notorious that no contrivance which had been devised until a very late period, at least for the purpose of such prevention, ever proved effectual; whether a canula invented by M. Reybard, and for which M. Sédillot appears to have conceived much affection, be destined to supply the desideratum, time must decide.* The curious point is that M. Sédillot seems to take much credit to himself for lauding a practice, of which the utility is so self-evident, that it must have been recommended (had it been considered capable of execution) by any one who reflected seriously on the subject. Dr. Walshe naturally records his approval of the plan, provided M. Reybard's instrument possess the properties assigned to it by its inventor.

This brings us to the question of the danger of pneumothorax. *Theoretically* considered, this appears far from inconsiderable; it is easy to imagine decomposition of pus, from the irritation of the pleural surface and pseudo-membranes and interference with the expansion of the lung, as being entailed by the entry of atmospheric air into the pleura, and as easy to suppose the progress of recovery seriously impeded thereby. But the truth is that *experience* by no means bears out these à priori notions, as, we are glad to observe, Dr. Walshe distinctly states; cases may be found in numbers in periodical publications and elsewhere demonstrating the innocuousness of pneumothorax, where the case has in other respects been one tending to a fortunate issue. And it is curious to note the contradictory notions upon the effects of pneumothorax to which à priori reasoning leads different observers,—an almost inevitable result in every instance where the phenomena either of health or disease are sought to be unravelled by its aid. M. Sédillot, although holding the entry of air to be in the main extremely detrimental, yet recognizes a positive advantage in its occurrence which he describes as follows: The atmospheric air having entered the pleura, "suppose the wound closed, inspiration will produce a kind of rarefaction of the fluids therein contained; but as this incipient vacuum is not filled up by air from without, it will impede the separation of the ribs, dilate the lung, prevent the depression of the diaphragm, and finally oppose the enlargement of the membranous sac lining the pleura. Now here is *an immense advantage in respect of the results of the operation.*"† On the other hand we find Dr. Williams, the importance of whose opinions upon such

* For an account of this canula, see Br. and For. Med. Rev., vol. XII., p. 251.

† The introduction of air into the chest of animals, provided it be not frequently repeated, appears from the results of M. Barthélemy's experiments, referred to by Dr. Walshe, to be perfectly harmless. The animals experimented on were, however, it must be remembered, healthy.

questions none will dispute, maintaining that "when air gets access, it tends to do mischief, whether the orifice remain open or closed; in the former case the air passing in and out prevents the lung from expanding, and constantly irritates the serous membranes; and if it be closed, the air admitted tends to engender more air by the decomposition which it causes in the remaining liquid, so that the pleura soon becomes as much distended as before the operation," (Lect. on Diseases of the Chest, No. xvii., Medical Gazette.) Who will not agree with us in recognizing the futility of argumentation, no matter how acute, if unsupported by extensive experimental evidence, while he has before him such illustrations of its results as that we now afford?

M. Sédillot next examines the subject of injections, dividing these into three classes, the diluent, the "modifying," and the oleo-vinous; we venture so to name the latter class as we confess ourselves unable to supply the author's omission of a title significative of the presumed mode of action of agents belonging thereto. The use of diluent injections, of which warm water is the best, is useful for the removal of pus of bad quality; but we cannot agree with M. Sédillot in his advocacy of the employment of "aromatic infusions, decoction of bark, tincture of aloes, nitrate of silver," for the purpose of altering the character of the suppurating surface, and believe Dr. Walshe's objections to such remedies, on the score of the want of all evidence from experience in their favour, justly urged. Respecting the third class M. Sédillot must speak for himself. "These injections," he says, "composed of a mixture of oil and wine, were according to the precepts of the Greek school, allowed to remain in the chest, and changed every twelve hours." [How could this be done without renewing the air in the pleura, a renewal which M. Sédillot pronounces to be so mischievous?] "They certainly deserve to be employed by surgeons of the present age, at the time recommended by the ancients, that is on the tenth day of the operation; for I have quoted cases which would appear to prove that a particular change does really occur at that period; and oil and wine forming a mild and gently exciting liquid, *might* beneficially modify the state of the parts." So much for oil and wine.

If we have exhibited any acerbity in our notice of M. Sédillot's book, this has been forced upon us by the egotism pervading his pages; there are certain styles of writing which would put the least irascible critic in the world out of temper, and M. Sédillot's is thus unfortunately characterized. There is, however, much in the volume besides the reprint of cases it contains, rendering it worthy of perusal, and we regret we are prevented from making some other of his dogmata, at least, the text for discussing the questions to which they refer. Dr. Hope's views seem to have been one-sided and imperfectly established, where they were found to have been old, and where new to have been deficient in solidity.

The detail with which we have noticed the essay of Dr. Walshe, is sufficient evidence of the estimation in which we hold that gentleman's production.

